# MCT4 Promotes Tumor Malignancy in F98 Glioma Cells

**DOI:** 10.1155/2021/6655529

**Published:** 2021-04-14

**Authors:** Anna Maria Reuss, Dominik Groos, Ali Ghoochani, Michael Buchfelder, Nicolai Savaskan

**Affiliations:** ^1^Laboratory for Translational Cell Biology & Neurooncology, Department of Neurosurgery, University Hospital Erlangen, Friedrich-Alexander University of Erlangen-Nuremberg (FAU), 91054 Erlangen, Germany; ^2^Institute of Physiology and Pathophysiology, Friedrich-Alexander University of Erlangen-Nuremberg (FAU), 91054 Erlangen, Germany

## Abstract

Monocarboxylate transporter 4 (MCT4, *SLC16A3*) is elevated under hypoxic conditions in many malignant tumors including gliomas. Moreover, MCT4 expression is associated with shorter overall survival. However, the functional consequences of MCT4 expression on the distinct hallmarks of cancer have not yet been explored at the cellular level. Here, we investigated the impact of MCT4 overexpression on proliferation, survival, cell death, migration, invasion, and angiogenesis in F98 glioma cells. Stable F98 glioma cell lines with MCT4 overexpression, normal expression, and knockdown were generated. Distinct hallmarks of cancer were examined using *in silico* analysis, various *in vitro* cell culture assays, and *ex vivo* organotypic rat brain slice culture model. Consistent with its function as lactate and proton exporter, MCT4 expression levels correlated inversely with extracellular pH and proportionally with extracellular lactate concentrations. Our results further indicate that MCT4 promotes proliferation and survival by altered cell cycle regulation and cell death mechanisms. Moreover, MCT4 overexpression enhances cell migration and invasiveness via reorganization of the actin cytoskeleton. Finally, MCT4 inhibition mitigates the induction of angiogenesis, suggesting that MCT4 also plays a crucial role in tumor-related angiogenesis. In summary, our data highlight MCT4*/SLC16A3* as a key gene for distinct hallmarks of tumor malignancy in glioma cells.

## 1. Introduction

Malignant gliomas are the most common primary malignant brain tumors with an increasing incidence of up to nine per 100,000 habitants over the last years [1, 2]. This brain tumor type is highly proliferative and shows an infiltrative growth pattern, accounting for the high recurrence rates in patients. Despite advances in surgical techniques and multimodal treatment regimens with radio-, chemo-, and immunotherapy [3–5], the prognosis for malignant glioma patients is very poor with a median survival time of approximately 15 months after diagnosis [6, 7].

The transmembrane protein MCT4, the respective gene termed *SLC16A3*, acts as an H^+^/lactate exporter in highly glycolytic tissues [8, 9]. Furthermore, MCT4 has been shown to be overexpressed in diverse types of neoplasia, among malignant gliomas [8–14]. Mechanistically, MCT4 is upregulated under hypoxia via binding of HIF-1*α* to two hypoxic response elements (HRE) within the *SLC16A3* promoter [15]. This process enables tumor cells, including those within the core of the heterogeneous tumor mass to survive, despite being at a distance from blood vessels that supply their metabolic needs [16, 17]. In the metabolic symbiosis model, these cells have been proposed to overexpress MCT4 to release high amounts of lactate produced during anaerobic glycolysis into the tumor microenvironment.

In line with this observation, elevated extracellular lactate concentrations up to 40 mM and corresponding low extracellular pH (pH_e_) have been found in solid tumors under hypoxic conditions [18–22]. Importantly, high lactate production and low pH_e_ have been related to tumor survival, progression, invasion, metastasis, chemoresistance, and recurrence [20, 23–28]. Furthermore, lactate has been proposed to stimulate angiogenesis through HIF-1*α*-dependent activation of the VEGF/VEGFR2 signaling pathway. In addition, the TGF*β*-mediated angiogenic pathway has been found to be induced by lactate [29–31]. MCT4 is reported to be coexpressed with VEGF family members in cervical adenocarcinomas [32]. Besides, upregulated MCT4 has been linked to altered tumor metabolism as well as to increased growth and survival in breast and pancreatic cancer [33, 34]. However, there has only been one previous functional study investigating MCT4 in glioblastoma (GBM) neurospheres, mainly focusing on tumor growth and survival dependent on the oxygen level [14].

In the present study, we examined the relationship between MCT4 expression, alterations in pH_e_, and extracellular lactate concentrations. We investigated the impact on tumor malignancy in F98 glioma cells using *in silico* analysis, *in vitro* cell culture assays, and *ex vivo* vascular organotypic glioma impact model (VOGIM) [35] by implanting F98 cells into rat brain slices.

For this purpose, we designed an MCT4 overexpression and knockdown/inhibition model to explore the functional consequences of MCT4 expression on cancer cell metabolism, survival, proliferation, migration, invasion potential, and angiogenesis as well as on cell cycle profiles and cell death mechanisms.

## 2. Materials and Methods

### 2.1. Cell Culture

Rat, human, and mouse glioma cell lines F98, U87, U251, and GL261 were obtained from ATCC/LGC-2397 (Wesel, Germany). All cell lines were cultured under standard humified conditions (37°C, 5% CO_2_) with DMEM (Biochrom, Berlin, Germany) supplemented with 10% FBS (Biochrom, Berlin, Germany), 1% penicillin/streptomycin (Biochrom, Berlin, Germany), and 1% GlutaMAX (Gibco, Darmstadt, Germany). For detachment, cells were treated with 0.05% trypsin (Biochrom, Berlin, Germany). HUVEC were cultured under standard humified conditions with Endothelial Cell Basal Medium with Endothelial Cell Growth Medium supplement mix (Promo Cell, Heidelberg, Germany). For detachment, cells were treated with StemPro® Accutase® (Gibco, Darmstadt, Germany) according to the manufacturer's instructions. All cells were evaluated under an inverted phase/fluorescence microscope (Olympus IX71, Hamburg, Germany) in the transmitted light path. Image acquisition was achieved via the Cell^F software (Olympus, Hamburg, Germany). To induce hypoxia, cells were treated with 200 *μ*M DFO (Sigma Aldrich, Taufkirchen, Germany). For MCT4 inhibition, cells were treated with 100 *μ*M pCMBS or 150 *μ*M phloretin (both from Sigma Aldrich, Taufkirchen, Germany). Cells were counted using the Cell Counter Z2 (Beckman Coulter, Brea, California, USA) according to the manufacturer's instructions.

### 2.2. Generation of Stable Cell Lines

For overexpression of the *MCT4/SLC16A3* gene, MCT4 cDNA (NM_001206950.1) was ligated into a peGFP-C1 vector (Clontech, Saint-Germain-en-Laye, France). For knockdown of MCT4 expression, siRNA 5′- and 3′-oligonucleotides targeting the MCT4 mRNA (Metabion, Martinsried, Germany; Supplementary Table S1) were ligated into a pSuper.neo + GFP vector (Oligoengine, Seattle, Washington, USA). For endogenous MCT4 expression within cells, the only pSuper.neo + GFP vector was used. DNA was augmented in the competent *E. coli* strain DH5*α* (Invitrogen, Darmstadt, Germany) and isolated with Nucleobond® gravity-flow columns according to manufacturer's instructions (Macherey-Nagel, Oensingen, Switzerland). For lipotransfection of F98 cells, 1 *μ*g DNA per sample was mixed with Opti-mem (Gibco, Darmstadt, Germany) and Rotifect (Carl Roth, Karlsruhe, Germany). For the selection of plasmid carrying cells, 1 mg/ml geneticin sulfate (G418; Biochrom, Berlin, Germany) within the culture medium was added immediately after transfection and reduced to a concentration of 700 *μ*g/ml, when cells showed stable expression. GFP-positive cells were further selected by FACS (Beckman Coulter MoFlo Legacy, Brea, California, USA).

### 2.3. pH_e_ and Lactate Concentration Measurements

Supernatants of cultured cells were collected for measurements. pH_e_ was determined via a pH meter (inoLab® WTW, Weilheim, Germany). Extracellular lactate concentrations were detected using Super GL (Hitado Diagnostic Systems, Moehnesee-Delecke, Germany) according to the manufacturer's instructions.

### 2.4. qRT-PCR

For RNA extraction from cells, the RNeasy® Mini Kit (QIAGEN, Hilden, Germany) was used according to the manufacturer's instructions. For RNA extraction from tissue, the MasterPure™ Complete DNA and RNA Purification Kit (Epicenter, Madison, Wisconsin, USA) was used according to the manufacturer's instructions. The concentration of purified RNA was determined via Nano Vue Plus (GE Healthcare, Little Chalfont, UK). For cDNA synthesis, the DyNAmo cDNA Synthesis Kit for qRT-PCR (Thermo Scientific, Schwerte, Germany) was used according to the manufacturer's instructions. For qRT-PCR, 1 *μ*g cDNA was applied and the ABsolute Blue QPCR SYBR Green Mix + separate ROX vial (Thermo Scientific, Schwerte, Germany) was used according to the manufacturer's instructions. Primers were designed using Beacon Designer 7 (PREMIER Biosoft, San Francisco, California, USA) and made by Metabion (Martinsried, Germany; Supplementary Table S2). Efficiencies of all primers were about 2 as assessed by calculated linear standard curves. *GAPDH* served as a housekeeping gene. Reactions were run in a Light Cycler 480 (Roche, Basel, Switzerland). Quantification was performed in Microsoft Excel (Microsoft, Redmond, Washington, USA) by calculating threshold values and fold changes relative to internal control (ΔΔCt method).

### 2.5. Immunofluorescence Staining

F98 cells were seeded onto glass platelets with a diameter of 20 mm (Thermo Scientific, Schwerte, Germany). After 48 h of culture, cells were fixed with 4% paraformaldehyde (PFA; Merck, Darmstadt, Germany) in PBS, followed by permeabilization and blocking with 0.2% TritonX-100 (Sigma Aldrich, Taufkirchen, Germany) and 3% FCS (Biochrom, Berlin, Germany) in PBS. All antibodies used in this study for immunofluorescence staining with the respective dilutions in blocking solution are listed in Supplementary Table S3. Pictures were taken with a Zeiss Imager A1 fluorescence microscope (Zeiss, Jena, Germany) at 40x magnification and an exposure time of 1 s for the green and 500 ms for the red channel for MCT4 staining and 300 ms for phalloidin staining, respectively. Image acquisition was achieved by using the software AxioVision (Zeiss, Jena, Germany). Mean fluorescence intensity was measured with the free software Fiji (NIH, Bethesda, Maryland, USA).

### 2.6. Flow Cytometry Analysis

For all experiments, the BD FACSCanto™ (BD Biosciences, Heidelberg, Germany) was used. For MCT4 expression analysis, GFP-positive cells were selected in the FITC 533/30 nm-A channel versus SSC. This cell population was displayed as a histogram using the FITC 533/30 nm-A parameter. Histograms of different samples were overlaid to show vector expression shifts. For cell cycle and cell death mechanisms' analysis, F98 cells were stained either with 7-AAD (see Supplementary Table S3) or with the Pacific Blue™ Annexin V Apoptosis Detection Kit with propidium iodide (PI) (Biolegend, London, UK) according to manufacturer's instructions. The FITC-A channel versus SSC was set to show GFP-positive cells. For cell cycle analysis, this cell population was then gated on PE-Cy5-W versus PE-Cy5-A and displayed as a histogram using PE-Cy5-A. When cell cycle profiles appeared, further gates were set to determine the percentage of distinct cell cycle phases. For cell death analysis, the GFP-positive population was gated on Pacific Blue-A versus Per-CP-A. Data were analyzed via the free Flowing software 2.5.1 (Cell imaging core, Turku Center for Biotechnology, Finland).

### 2.7. MTT Assay

The MTT assay is widely used for assessing cell viability as described previously [36]. Briefly, 5,000 and 3,000 F98 cells were seeded per 96-well (Greiner BioOne, Frickenhausen, Germany) and cultured for 48 h and 72 h, respectively. Then, 50 *μ*g 3-(4, 5-dimethylthiazol-2-yl)-2, 5-diphenyltetrazolimbromide (MTT; Carl Roth, Karlsruhe, Germany) was added to each well. After incubation for 4 h, cells were lysed by shaking in isopropanol with 37% HCl. Spectrophotometric measurement was performed via an SLT Spectra II microplate reader (SLT Labinstruments, Crailsheim, Germany). To assess ferroptosis as described previously [37], cells were additionally treated with 10 *μ*M erastin and 1 *μ*M ferrostatin (both from Sigma Aldrich, Taufkirchen, Germany) per well before culturing.

### 2.8. Scratch Assay

The scratch assay is widely used for evaluating migration [38]. Briefly, 25,000 F98 cells were seeded per 24-well (Becton Dickinson Labware) and cultured up to confluence. The medium was replaced and a scratch was drawn via a yellow pipette tip. At 0 h as well as after 12 h and 24 h, pictures were taken with an Olympus IX71 microscope at 10x magnification in the transmitted light path. Image acquisition was achieved via the Cell^F software of the microscope (Olympus, Hamburg, Germany), and scratch length measurement was performed with the free software Fiji (NIH, Bethesda, Maryland, USA).

### 2.9. Colony Formation Assay

The colony formation assay investigates invasion in a 3D cell culture model [39]. Briefly, 20,000 F98 cells were seeded per 6-well and cultured in a 3D environment formed by a mixture of agarose and DMEM with additives. After 7 d, pictures of forming colonies were taken with an Olympus IX71 microscope at 10x magnification in the transmitted light path. Image acquisition was achieved via the Cell^F software of the microscope (Olympus, Hamburg, Germany), and analysis was performed with the free software Fiji (NIH, Bethesda, Maryland, USA).

### 2.10. Endothelial Cell Tube Formation Assay

The endothelial cell tube formation assay [40] was performed with HUVEC. Briefly, a mixture of 70% Matrigel (BD Biosciences) and 30% DMEM without additives was added per 96-well and incubated at 37°C and 5% CO_2_ for 1 h. After polymerization, 10 000 HUVEC were seeded per 96-well onto the gel bottom. Cells were treated with conditioned media of F98 cells and endothelial cell media in a ratio of 1 : 1. As a positive control, HUVEC were treated with 10 mM lactate in endothelial cell media. After 12 h in 3D cell culture, pictures were taken with an Olympus IX71 microscope at 10x magnification in the transmitted light path. Image acquisition was achieved via the Cell^F software of the microscope (Olympus, Hamburg, Germany), and analysis was performed with the free software Fiji (NIH, Bethesda, Maryland, USA).

### 2.11. VOGIM Slice Culture

VOGIM slice culture with P6 old Wistar rats (Charles River Laboratories, Sulzfeld, Germany) was performed as described previously [35]. For tumor implantation, slices were randomly distributed over distinct groups. Only slices of superior quality were used as determined via PI staining (see Supplementary Table S3). To measure cell death, this procedure was repeated directly after tumor implantation as well as after 48 h. Pictures were taken at 4x magnification (Olympus IX71, Hamburg, Germany) and an exposure time of 500 ms for the green and 50 ms for the red channel. Image acquisition was achieved via the Cell^F software of the microscope (Olympus, Hamburg, Germany), and analysis was performed using the free software Fiji (NIH, Bethesda, Maryland, USA). After 5 d, slices were fixed in PBS with 4% PFA and 1.2% picric acid, pH = 7.4 with NaOH (all reagents from Merck, Darmstadt, Germany), and stained for laminin (see Supplementary Table S3) to display blood vessels via the protocol described above. Pictures of embedded slices were taken with a Zeiss Imager A1 fluorescence microscope (Jena, Germany) at 10x magnification and an exposure time of 1.5 s for the green and 500 ms for the red channel. Image acquisition was achieved with the software AxioVision of the microscope (Zeiss, Jena, Germany), and analysis was performed using the free software Fiji (NIH, Bethesda, Maryland, USA).

### 2.12. Data Analysis

For *in silico* analyses, the databases GENT2 [41], IVY Glioblastoma Atlas Project [42], and the STRING database [43] were used. Statistical analyses were performed with Microsoft Excel, the licensed software MATLAB, and the licensed software GraphPad Prism 5 (La Jolla, California, USA). Data are represented as mean ± standard errors of the mean (SEM) and *n* gives the number of independent experiments. Normality has been tested using the Shapiro-Wilk normality test. Comparisons were then evaluated statistically using Student's *t*-test with or without FDR correction and ANOVA in a one-way or two-way model with Tukey's or Bonferroni posttests. Significance was assumed for *p* < 0.05.

### 2.13. Schematic Illustrations

Schematic illustrations shown in this manuscript were created with the free software version of BioRender (Toronto, Canada).

## 3. Results

### 3.1. MCT4 Overexpression under Hypoxia Correlates with Clinical Outcome in GBM Patients

**Figure 1:**
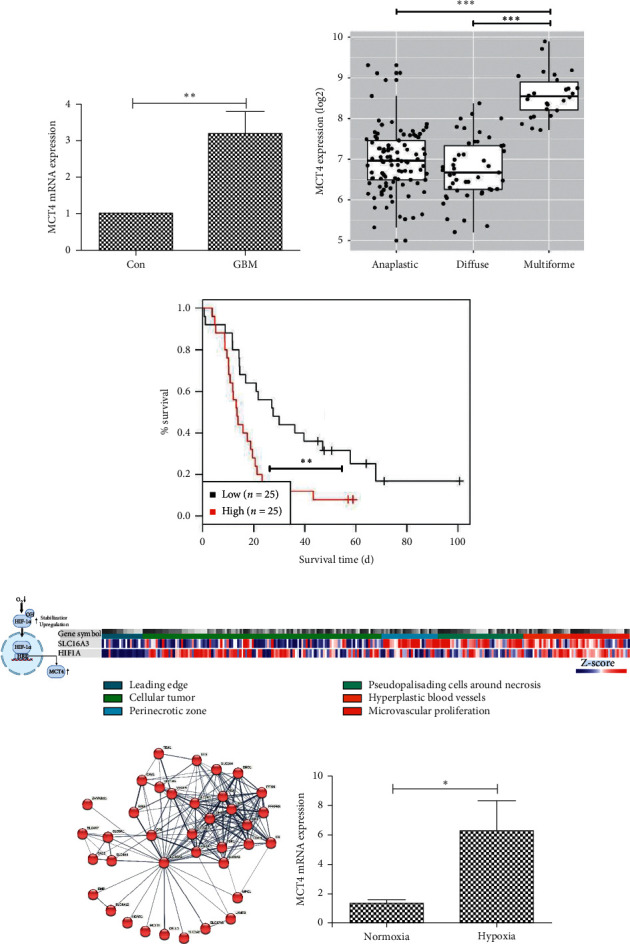
Endogenous MCT4 expression in GBM and F98 glioma cells. (a) Relative MCT4 mRNA expression in GBM normalized to con as determined by qRT-PCR. Statistical analysis was performed by Student's *t*-test (^*∗∗*^*p* < 0.01, mean ± SEM, *n* = 7). (b) MCT4 expression in different glioma subtypes. Data were obtained from the GENT2 database. Statistical analysis was performed by Student's *t*-test with FDR correction for multiple testing (^*∗∗∗*^*p* < 0.001, mean ± SEM, *n* > 26). (c) Survival curves of patients with different MCT4 expression levels in GBM. High and low expressions are defined as above and below the median expression, respectively. Data were obtained from the GENT2 database. Statistical analysis was performed by log-rank test (^*∗∗*^*p* < 0.01, mean ± SEM, *n* = 25). (d) Scheme of MCT4 regulation by hypoxia via HIF-1*α* and gene expression analysis of MCT4/*SLC16A3* and HIF-1*α*/*HIF1A* in histological GBM compartments, shown as normalized gene-level FPKM values (*n* = 278). Data were obtained from the IVY Glioblastoma Atlas Project database. (e) Network analysis for highest interactors with MCT4/*SLC16A3*. Shown are all connections between interactors. Data were obtained from the STRING database. (f) Relative MCT4 mRNA expression in F98 wild-type (wt) cells cultured under hypoxic conditions and normalized to normoxic cell culture conditions as determined by qRT-PCR. Statistical analysis was performed with Student's *t*-test (^*∗*^*p* < 0.05, mean ± SEM, *n* = 3).

MCT4 has been shown to be overexpressed in diverse neoplasia, including malignant gliomas [8–14]. Therefore, we first examined MCT4 expression via qRT-PCR in tumor tissue from our GBM patient cohort. MCT4 mRNA was upregulated about 3-fold in GBM compared to nonneoplastic control tissue (Figure 1(a)). To further elucidate MCT4 expression in GBM, we performed *in silico* analysis of MCT4 expression in brain tumors of different histological glioma grades obtained from the GENT2 database [41]. MCT4 showed enhanced expression in WHO grade IV GBM compared to WHO grade II diffuse and WHO grade III anaplastic astrocytoma (Figure 1(b)). Interestingly, GBM from patients with high MCT4 expression exhibited lower survival rates compared to those with low MCT4 expression (Figure 1(c)).

MCT4 is upregulated under hypoxic conditions via binding of HIF-1*α* to two HRE within the promotor of the MCT4 gene *SLC16A3* (Figure 1(d)) [15]. Interestingly, *in silico* analysis of MCT4/*SLC16A3* and HIF-1*α*/*HIF1A* expression in microdissected GBM tumor compartments obtained from the IVY Glioblastoma Atlas Project database [42] revealed an expression gradient for both genes from the leading edge towards the tumor center in most tumor specimens (Figure 1(d)). Here, MCT4/*SLC16A3* expression was particularly high in perinecrotic zones and in the hyperplastic/microvascular proliferation areas, both histological criteria that distinguish GBM from anaplastic astrocytoma [44]. Overall, most differentially expressed genes (DEGs) in the perinecrotic and hyperplastic/microvascular proliferation zones were upregulated compared to the leading edge (Supplementary Figure S1A). Network analysis for the highest interactors with MCT4/*SLC16A3* revealed HIF-1*α*/*HIF1A* as one of the nearest neighbors (Figure 1(e)).

Since MCT4 overexpression in GBM under hypoxia seemed to have substantial clinical relevance, we wanted to study its functional consequences in an overexpression and knockdown/inhibition glioma cell model. For genetic manipulation of glioma cells, we first examined via qRT-PCR the endogenous MCT4 expression levels in distinct glioma cell lines from different species (Supplementary Figure S1B). We selected F98 glioma cells with the lowest endogenous MCT4 mRNA expression for genetic manipulation so that genetically induced MCT4 overexpression would not be disturbed by already high endogenous MCT4 expression levels. To further characterize the regulation of endogenous MCT4 expression in these cells, we induced hypoxia by deferoxamine (DFO) treatment and determined MCT4 mRNA expression via qRT-PCR. Of note, MCT4 mRNA expression increased about 6-fold in F98 cells under hypoxia compared to normoxia (Figure 1(f)). Therefore, MCT4 seems to be upregulated in GBM under hypoxic conditions via HIF-1*α*, mainly in the perinecrotic and hyperplastic/microvascular proliferation tumor zones. To study the functional consequences of MCT4 expression, F98 cells proved to be an appropriate cell line.

### 3.2. MCT4 Expression Correlates with Extracellular pH and Lactate Concentration in F98 Glioma Cells

**Figure 2:**
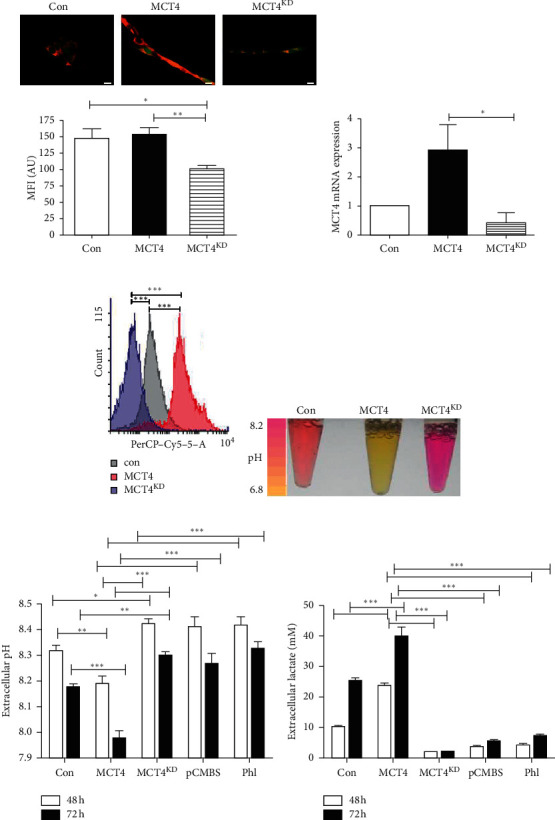
MCT4 overexpression and knockdown phenotype in F98 glioma cells. (a) Immunocytological staining for MCT4 expression (red) in GFP-positive con, MCT4, and MCT4^KD^ F98 cells (green). Scale bar represents 10 *μ*m. Quantification of mean fluorescence intensity (MFI) of the red channel is shown below. Statistical analysis was performed by one-way ANOVA with Tukey's posttest (^*∗*^*p* < 0.05; ^*∗∗*^*p* < 0.01, mean ± SEM, *n* = 11). (b) MCT4 mRNA expression ratios in con, MCT4, and MCT4^KD^ F98 cells, normalized to con, as determined by qRT-PCR. Statistical analysis was performed by one-way ANOVA with Tukey's posttest (^ns^*p* > 0.05; ^*∗*^*p* < 0.05, mean ± SEM, *n* = 5). (c) MCT4 protein expression in con, MCT4, and MCT4^KD^ F98 cells determined by flow cytometry analysis. Statistical analysis was performed by one-way ANOVA with Tukey's posttest (^*∗∗∗*^*p* < 0.001, mean ± SEM, *n* = 3). (d) Supernatants of con, MCT4, and MCT4^KD^ F98 cells cultured for 72 h. (e) pH_e_ measured within the supernatants of con, MCT4, and MCT4^KD^ F98 cells as well as MCT4 cells treated with 100 *μ*M pCMBS or 150 *μ*M Phl and cultured for 48 h and 72 h. Statistical analysis was performed by two-way ANOVA with Bonferroni posttest (^*∗*^*p* < 0.05, ^*∗∗*^*p* < 0.01, and ^*∗∗∗*^*p* < 0.001, mean ± SEM, *n* = 5). (f) Extracellular lactate concentrations measured in the supernatants of con, MCT4, and MCT4^KD^ F98 cells as well as MCT4 cells treated with 100 *μ*M pCMBS or 150 *μ*M Phl and cultured for 48 h and 72 h. Statistical analysis was performed by two-way ANOVA with Bonferroni posttest (^*∗∗∗*^*p* < 0.001, mean ± SEM, *n* = 3).

Stable cell lines with endogenous MCT4 expression only (con), MCT4 overexpression (MCT4), and MCT4 knockdown (MCT4^KD^) were generated as described in the Materials and Methods section. All three tested siRNAs transiently knocked down MCT4 mRNA expression by about 50% (Supplementary Figure S2A). siRNA#2 displayed the highest reduction and was thus chosen to generate the stable MCT4 mRNA knockdown cell line (MCT4^KD^). Staining of stably transfected GFP-positive F98 glioma cells with anti-MCT4 antibody showed MCT4 to mainly localize in the outer cellular rim, consistent with its function as a transporter within the plasma membrane (Figure 2(a)). Furthermore, MCT4 cells displayed slightly enhanced fluorescence intensity compared to con, whereas fluorescence intensity was significantly reduced in MCT4^KD^ cells, indicating differences in MCT4 expression in the respectively transfected cells. Determination of MCT4 mRNA expression in stably transfected F98 glioma cells via qRT-PCR revealed approximately 3-fold upregulation of MCT4 in MCT4 cells compared to con. In contrast, MCT4 was downregulated about half in MCT4^KD^ cells (Figure 2(b)). Although overexpression was not significant at the mRNA level as assessed by a rather stringent statistical test, 3-fold upregulation can be considered sufficient for stably transfected cells. Importantly, this finding mirrored the enhanced MCT4 expression levels seen in GBM patients (Figure 1(c)). Furthermore, the observed differences in MCT4 expression were significant between all groups at the protein level determined by flow cytometry analysis (Figure 2(c)).

MCT4 acts as a lactic acid exporter by lactate and H^+^ symport [9]. Consistent with this function was the finding that the supernatants of MCT4 F98 glioma cells cultured in DMEM with phenol red as pH indicator were yellow, indicating a lower pH_e_ than those of con, which were pinkish to orange (Figure 2(d)). Vice versa, the supernatants of MCT4^KD^ cells showed a deeply pink color, indicating the highest pH_e_. To further explore this observation, we measured the pH_e_ of stably transfected F98 glioma cells after culture for 48 h and 72 h. To validate the knockdown and to exclude off-target effects, an additional approach was chosen to inhibit the MCT4 function. We treated MCT4 F98 glioma cells with the two widely applied MCT4 inhibitors *p*-chloromercuribenzene sulfonate (pCMBS) and phloretin (Phl) [45]. Nontoxic treatment concentrations for F98 cells were determined via MTT assay (Supplementary Figures S2B and S2C). Indeed, pH_e_ of MCT4 F98 glioma cells was significantly lower after 48 h in culture than pH_e_ of con (Figure 2(e)). In contrast, the supernatants of MCT4^KD^ cells and inhibitor-treated MCT4 cells exhibited significantly higher pH_e_ compared to con and to MCT4, respectively. After 72 h, the pH_e_ of all F98 glioma cells decreased, a physiological phenomenon observed in cells cultured for an extended period. However, differences between the respectively transfected cells described previously were even stronger than after 48 h. In accordance with the lower pH_e_ in MCT4 cells, extracellular lactate concentration should be increased. Therefore, we measured extracellular lactate concentrations in the supernatants of stably transfected and inhibitor-treated F98 glioma cells after culture for 48 h and 72 h. As expected, extracellular lactate concentration was higher for MCT4 cells than for con and even more than for MCT4^KD^ and inhibitor-treated MCT4 cells after 48 h as well as after 72 h (Figure 2(f)).

With these experiments, we have shown that MCT4 expression has functional effects. Therefore, this model has allowed us to study the functional consequences of MCT4 expression in F98 glioma cells.

### 3.3. MCT4 Overexpressing F98 Glioma Cells Display Typical Characteristics of Tumor Malignancy

**Figure 3:**
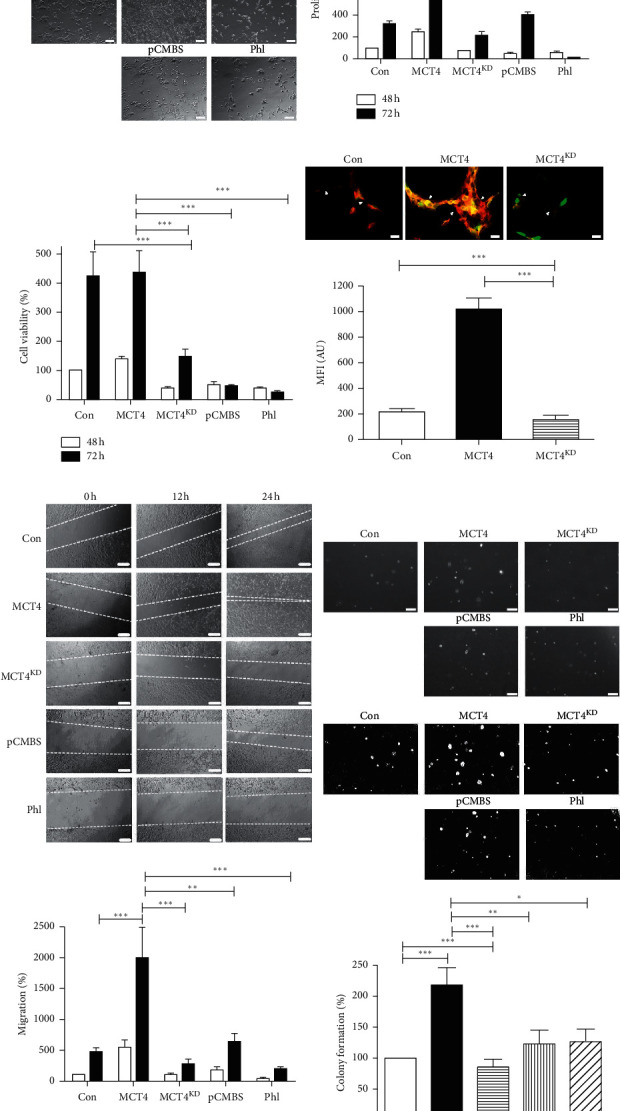
Malignancy in MCT4 overexpression and knockdown F98 glioma cells. (a) Representative images of cell density of con, MCT4, and MCT4^KD^ F98 cells as well as MCT4 cells treated with 100 *μ*M pCMBS or 150 *μ*M Phl. Scale bar represents 200 *μ*m. (b) Proliferation of con, MCT4, and MCT4^KD^ F98 cells as well as MCT4 cells treated with 100 *μ*M pCMBS or 150 *μ*M Phl and cultured for 48 h and 72 h as determined by cell counting. Data were normalized to con after 48 h. Statistical analysis was performed by two-way ANOVA with Bonferroni posttest (^*∗∗∗*^*p* < 0.001, mean ± SEM, *n* = 3). (c) Cell viability of con, MCT4, and MCT4^KD^ F98 cells as well as MCT4 cells treated with 100 *μ*M pCMBS or 150 *μ*M Phl and cultured for 48 h and 72 h as determined by MTT assay. Data were normalized to con after 48 h Statistical analysis was performed by two-way ANOVA with Bonferroni posttest (^*∗∗∗*^*p* < 0.001, mean ± SEM, *n* = 3). (d) Actin cytoskeleton morphology in con, MCT4, and MCT4^KD^ F98 cells (green), visualized by phalloidin staining (red). Arrows indicate cytoskeleton networks between cells. Scale bar represents 20 *μ*m. Quantification of MFI of the red channel is shown below. Statistical analysis was performed by one-way ANOVA with Tukey's posttest (^*∗∗∗*^*p* < 0.001, mean ± SEM, *n* ≥ 3). (e) Migration of con, MCT4, and MCT4^KD^ F98 cells as well as MCT4 cells treated with 100 *μ*M pCMBS or 150 *μ*M Phl after 12 h and 24 h as determined by scratch assay (dashed lines). Scale bar represents 200 *μ*m. Quantification was performed by measuring the scratch length with Fiji and normalizing it to 0 h and con. Statistical analysis was performed by two-way ANOVA with Bonferroni posttest (^*∗∗*^*p* < 0.01; ^*∗∗∗*^*p* < 0.001, mean ± SEM, *n* = 3). (f) Colony formation of con, MCT4, and MCT4^KD^ F98 cells as well as MCT4 cells treated with 100 *μ*M pCMBS or 150 *μ*M Phl after 7 d in 3D cell culture. Images in the upper row depict raw data, whereas images in the row below are postprocessed with Fiji. Scale bar represents 200 *μ*m. Quantification was performed with Fiji and normalized to con. Statistical analysis was performed by one-way ANOVA with Bonferroni posttest (^*∗*^*p* < 0.05, ^*∗∗*^*p* < 0.01, and ^*∗∗∗*^*p* < 0.001, mean ± SEM, *n* = 3).

Interestingly, during cell culture, we observed that MCT4 F98 glioma cells reached confluence faster than con, whereas MCT4^KD^ cells were much less dense (Figure 3(a)). Findings were similar when MCT4 F98 glioma cells were treated with the inhibitor pCMBS and phloretin. To further explore this phenomenon, we counted distinct groups of stably transfected and inhibitor-treated F98 glioma cells after culture for 48 h and 72 h. Indeed, MCT4 cells grew faster than con and MCT4^KD^ cells after 48 h and even more after 72 h. In contrast, inhibitor treatment reduced the proliferation of MCT4 cells after 48 h and 72 h (Figure 3(b)).

Besides uncontrolled proliferation, the hallmarks of cancer cells comprise increased survival as well as migration and invasion [46]. Hence, we further aimed to study these characteristics in relation to MCT4 expression. After culture for 48 h, metabolism-dependent cell viability determined via MTT assay was increased in MCT4 F98 glioma cells in comparison with con, MCT4^KD^, and inhibitor-treated MCT4 cells (Figure 3(c)). After 72 h, MCT4 inhibition significantly decreased cell viability.

Cytoskeleton morphology is altered in malignant cells, enabling detachment from the cell layer followed by migration, a precondition for invasion and metastasis [47]. To study actin cytoskeleton morphology in transfected glioma cells, we performed phalloidin staining. MCT4 F98 glioma cells showed more actin fibers with larger microfilament networks than con and even more than MCT4^KD^ cells (Figure 3(d)). To examine the migration potential of these cells, we conducted a scratch assay. Consistent with the observations in phalloidin staining, MCT4 F98 glioma cells migrated faster into the scratch than con, MCT4^KD^, and inhibitor-treated MCT4 cells after 12 h and even more after 24 h (Figure 3(e)).

The potential of tumor cells to form colonies is another prerequisite for invasion [39]. Therefore, we performed a colony formation assay in a 3D cell culture environment. MCT4 F98 glioma cells formed more and larger colonies, whereas con, MCT4^KD^, and inhibitor-treated MCT4 cells formed less and smaller ones (Figure 3(f)). Matrix metalloproteinases (MMPs) are known to play a crucial role during invasion of diffuse gliomas [48]. We thus performed *in silico* expression analysis for different MMPs in the perinecrotic and hyperplastic/microvascular proliferation zones, in which MCT4/*SLC16A3* was highly overexpressed. Interestingly, distinct MMPs clustered into subsets that were predominantly upregulated either in the perinecrotic or in hyperplastic/microvascular proliferation zones (Supplementary Figure S3A). Furthermore, epithelial-mesenchymal transition (EMT) has been proposed to play an important role during the invasion of gliomas [49, 50]. Interestingly, MCT4/*SLC16A3* was upregulated in 38% of GBMs with mesenchymal molecular subtype, but only 23–25% in the ones with other molecular subtypes (Supplementary Figure S3B). We investigated the expression profiles of the 22 most EMT-associated genes in the perinecrotic and hyperplastic/microvascular proliferation zones, in which MCT4/*SLC16A3* was highly overexpressed. Most EMT-associated genes were found in the hyperplastic/microvascular proliferation tumor zones (Supplementary Figure S3C). However, the correlation of expression patterns was not evident in all GBM samples.

In summary, these results illustrate that MCT4 overexpressing F98 glioma cells display typical characteristics of tumor malignancy, namely, excessive proliferation, cell survival, and altered cytoskeleton morphology as well as enhanced migration and invasive potential.

### 3.4. MCT4 Inhibition Reduces the Induction of Angiogenesis

**Figure 4:**
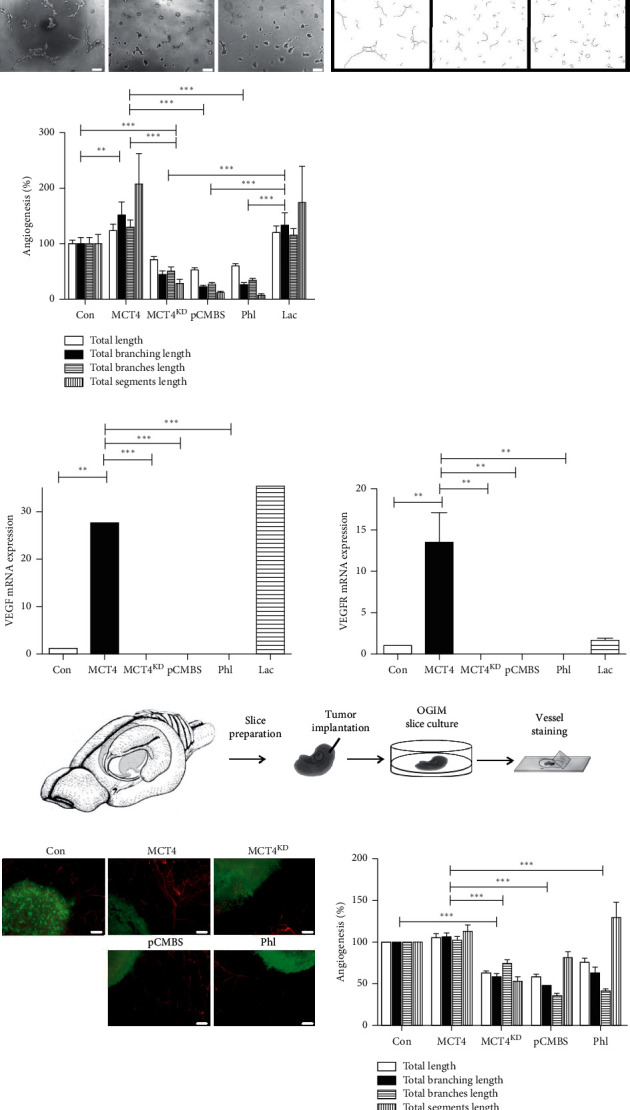
Angiogenesis in MCT4 overexpression and knockdown F98 glioma cells. (a) Tube formation of HUVEC in 3D cell culture treated with the conditioned media of con, MCT4, and MCT4^KD^ F98 cells as well as with the conditioned media of MCT4 cells treated with 100 *μ*M pCMBS, 150 *μ*M Phl, or 10 mM Lac. Images on the left depict raw data, whereas images on the right are postprocessed with Fiji. Scale bar represents 200 *μ*m. Quantification of tube formation parameters for the assessment of angiogenesis was performed with Fiji. Data were normalized to con. Statistical analysis was performed by two-way ANOVA with Bonferroni posttest (^*∗∗*^*p* < 0.01; ^*∗∗∗*^*p* < 0.001, mean ± SEM, *n* = 3). (b) VEGF and VEGFR mRNA expression ratios in HUVEC treated with the conditioned media of con, MCT4, and MCT4^KD^ F98 cells as well as with the conditioned media of MCT4 cells treated with 100 *μ*M pCMBS, 150 *μ*M Phl, or 10 mM Lac, as determined by qRT-PCR. Data were normalized to con. Statistical analysis was performed by one-way ANOVA with Tukey's posttest (^*∗∗*^*p* < 0.01; ^*∗∗∗*^*p* < 0.001 mean ± SEM, *n* = 3). (c) Scheme of the procedure for VOGIM slice culture. (d) Blood vessel sprouting in VOGIM slices 5 d after tumor implantation with con, MCT4, and MCT4^KD^ F98 cells (green) as well as MCT4 cells treated with 100 *μ*M pCMBS or 150 *μ*M Phl (green), visualized by antilaminin staining (red). Quantification of blood vessel sprouting parameters for the assessment of angiogenesis was performed with Fiji. Data were normalized to con. Statistical analysis was performed by two-way ANOVA with Bonferroni posttest (^*∗∗∗*^*p* < 0.001, mean ± SEM, *n* = 9).

A further hallmark of malignancy is angiogenesis, which is essential for oxygen and metabolic supply, and thus fosters tumor growth after a certain tumor size has been reached [46]. To study this characteristic *in vitro*, we performed a tube formation assay with HUVEC treated with the conditioned media of stably transfected and inhibitor-treated F98 glioma cells in a 3D cell culture environment. As a positive control, HUVEC were treated with 10 mM lactate (Lac). To exclude the toxic effects of treatments, HUVEC were first treated with pCMBS, phloretin, or lactate alone. No reduction in tube formation was detected for pCMBS or lactate treatment compared to untreated control (ctrl) (Supplementary Figure S4A). Phloretin showed a decrease in two angiogenic parameters compared to untreated control, but not compared to lactate treated cells. Therefore, phloretin was chosen as a secondary inhibitor. Then, HUVEC were treated with the conditioned media of F98 glioma cells. Compared to con, HUVEC treated with the conditioned media of MCT4 F98 glioma cells showed larger tube formation and more branching akin to lactate treatment (Figure 4(a)). In contrast, MCT4 inhibition via knockdown or pCMBS and phloretin treatment substantially decreased tube formation.

Consistent with this finding, HUVEC treated with the conditioned media of MCT4 F98 glioma cells exhibited increased VEGF and VEGFR mRNA expression levels compared to those treated with the conditioned media of con, MCT4^KD^, and inhibitor-treated MCT4 F98 glioma cells (Figure 4(b)).

To further validate these results in an *ex vivo* model, we performed VOGIM slice culture. Briefly, hippocampal slices were cut from the brains of 6-days old Wistar rats using a vibratome, implanted with stably transfected F98 glioma cells of distinct groups, among two treated with the inhibitors pCMBS and phloretin, and cultured under humified conditions (Figure 4(c)). To exclude toxic effects of the treatments on the slices, native brain slices without tumor implantation were treated with pCMBS, phloretin, or lactate and stained for cell death intensity via PI after 48 h as well as for blood vessels after 5 d in culture. Compared to the untreated control, none of the treatments led to a significant increase in cell death intensity (Supplementary Figure S4B). Furthermore, angiogenic parameters increased under lactate treatment as expected but did not decrease under inhibitor treatment (Supplementary Figure S4C). In contrast, phloretin seemed to enhance angiogenesis compared to the untreated control. After 5 d in culture, tumor implanted brain slices were fixed and stained for vascular morphology. In comparison to con, MCT4 gliomas were surrounded by larger and more branched blood vessels, albeit minimally (Figure 4(d)). However, inhibition of MCT4 expression via knockdown or inhibitor treatment significantly reduced angiogenic parameters.

These results indicate that MCT4 inhibition impedes the induction of angiogenesis, another crucial hallmark of tumor malignancy, in F98 glioma cells *in vitro* as well as in an *ex vivo* slice culture model, which more closely reflects the *in vivo* situation.

### 3.5. MCT4 Promotes Tumor Growth in *Ex Vivo* VOGIM Slice Culture

**Figure 5:**
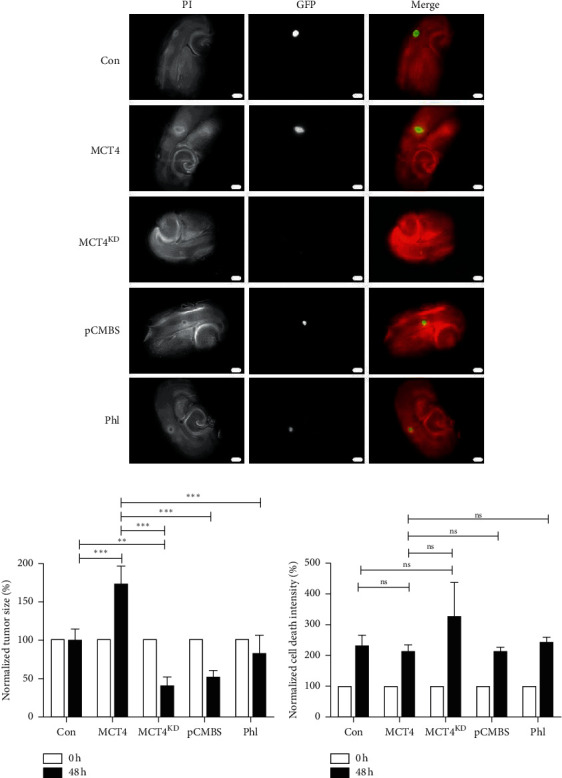
Tumor growth and cell death in MCT4 overexpression and knockdown F98 VOGIM slices. (a) VOGIM slices implanted with con, MCT4, and MCT4^KD^ F98 cells (green) as well as MCT4 cells treated with 100 *μ*M pCMBS or 150 *μ*M Phl (green) and stained with PI (red) directly after and 48 h after tumor implantation and treatment. (b) Quantification of tumor growth in VOGIM slices was performed with Fiji. Data were normalized to con. Statistical analysis was performed by two-way ANOVA with Bonferroni posttest (^*∗∗*^*p* < 0.01; ^*∗∗∗*^*p* < 0.001, mean ± SEM, *n* = 9). (c) Quantification of tumor death in VOGIM slices was performed with Fiji. Data were normalized to con. Statistical analysis was performed by two-way ANOVA with Bonferroni posttest (^ns^*p* > 0.05, mean ± SEM, *n* = 9).

To validate the *in vitro* results for proliferation and survival in an *ex vivo* slice culture model, we measured tumor growth and tumor death in VOGIM slices during culture. Normalized to the time point of tumor implantation (0 h), MCT4 gliomas showed an increase in tumor growth after 48 h, whereas the tumor size of MCT4^KD^ and inhibitor-treated MCT4 gliomas decreased (Figures 5(a) and 5(b)).

Tumor death intensity determined via PI staining was not significantly altered between groups, which is likely due to the overall increase in PI staining, masking the expected differences (Figure 5(c)).

These data support the *in vitro* obtained results that MCT4 increases tumor growth significantly also in *ex vivo* VOGIM slice culture. There was a trend towards MCT4 overexpression to protect from tumor death, despite being not statistically significant in the *ex vivo* slice culture model.

### 3.6. MCT4 Knockdown Alters Cell Cycle Profile and Cell Death Mechanisms

**Figure 6:**
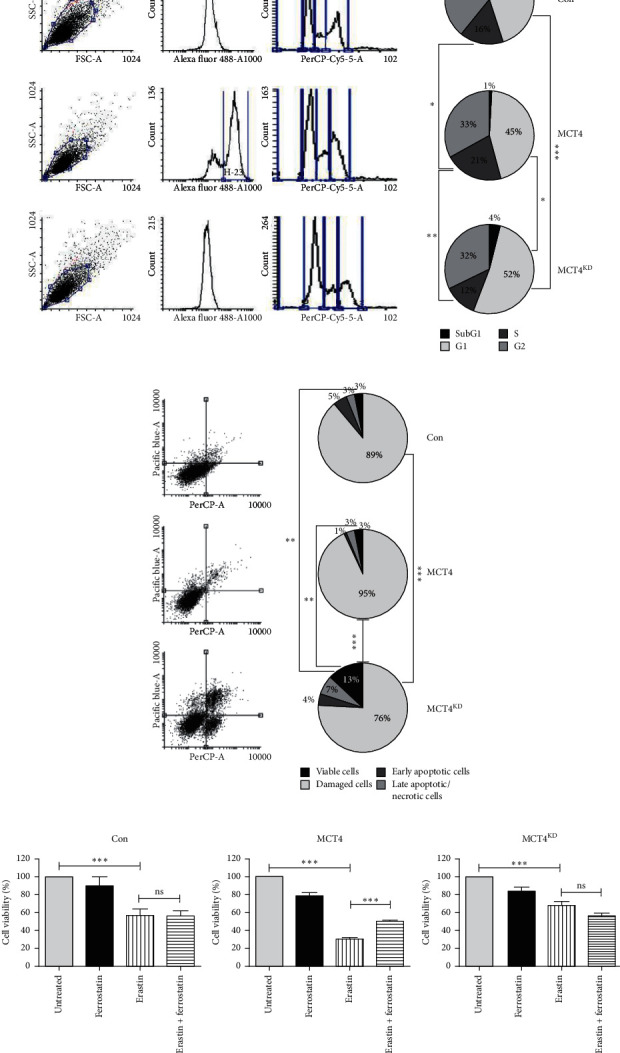
Cell cycle profiles and cell death mechanisms in MCT4 overexpression and knockdown F98 glioma cells. (a) Cell cycle analysis of con, MCT4, and MCT4^KD^ F98 cells, as determined by flow cytometry analysis. Statistical analysis was performed by two-way ANOVA with Bonferroni posttest (^*∗*^*p* < 0.05, ^*∗∗*^*p* < 0.01, and ^*∗∗∗*^*p* < 0.001, mean ± SEM, *n* = 3). (b) Cell death mechanisms in con, MCT4, and MCT4^KD^ F98 cells, as determined by flow cytometry analysis. Viable cells are defined as both PI- and Annexin V-negative, damaged cells are defined as PI-positive and Annexin V-negative, apoptotic cells are defined as PI-negative and Annexin V-positive, and late apoptotic/necrotic cells are defined as both PI- and Annexin V-positive. Statistical analysis was performed by two-way ANOVA with Bonferroni posttest (^*∗∗*^*p* < 0.01; ^*∗∗∗*^*p* < 0.001, mean ± SEM, *n* = 3). (c) Ferroptosis in con, MCT4, and MCT4^KD^ F98 cells treated with 10 *μ*M erastin and 1 *μ*M ferrostatin, as determined by MTT assay. Data were normalized to untreated controls. Statistical analysis was performed by one-way ANOVA with Bonferroni posttest (^ns^*p* > 0.05; ^*∗∗∗*^*p* < 0.001, mean ± SEM, *n* = 3).

Our aim was to further investigate the role of MCT4 in tumor death since the underlying mechanism was unclear. Possible explanations for increased proliferation and cell survival are resistance to apoptosis as well as uncontrolled entrance into the active cell cycle, which are further hallmarks of cancer cells [46]. Therefore, we examined cell cycle profiles and distinct mechanisms of cell death under MCT4 overexpression and knockdown in stably transfected F98 glioma cells. Negative controls for gating are shown in Supplementary Figures S5A and S5B. Cell cycle profiling via flow cytometry analysis showed a significantly larger amount of MCT4 F98 glioma cells to be in the *S*-phase compared to con and MCT4^KD^ cells (Figure 6(a)). In contrast, MCT4^KD^ cells remained particularly within the *G*1-phase. The fraction of dead cells (*SubG*1) was low in all groups, although it was highest in MCT4^KD^ and lowest in MCT4.

To explore the distinct cell death mechanisms within these cells, we stained for Annexin V and PI and performed flow cytometry analysis. With 93%, MCT4 showed the highest fraction of viable cells, MCT4^KD^ with 76% showed the lowest of all three groups (Figure 6(b)). Conversely, MCT4^KD^ had the largest proportions of early apoptotic and late apoptotic/necrotic cells compared to con and MCT4.

Recently, an additional iron-dependent mechanism of cell death called ferroptosis was proposed [37]. To investigate whether ferroptosis plays a role in MCT4-dependent cell death, we conducted an MTT assay combined with erastin treatment and subsequent rescue via ferrostatin, a well-known inhibitor of ferroptosis [37]. Compared to untreated controls, erastin significantly reduced cell viability in all three groups (Figure 6(c)). However, only in MCT4 F98 glioma cells, ferrostatin was able to partially rescue cell viability, suggesting ferroptosis to play a certain role exclusively when MCT4 is upregulated.

In summary, the results described above suggest that MCT4 promotes cell cycle progression and augments cell survival by protecting tumor cells from cell death, particularly from late apoptosis/necrosis. Moreover, ferroptosis is proposed to occur only in the presence of MCT4 overexpression, but not when MCT4 is expressed at baseline level or is silenced.

## 4. Discussion

MCT4/*SLC16A3* has been shown to be overexpressed in many malignant tumors including gliomas under hypoxic conditions within the tumor center [8–14]. In our GBM patient cohort, we confirmed that MCT4 was upregulated about 3-fold. Using *in silico* analysis, we further showed that MCT4 was significantly overexpressed in WHO grade IV GBM compared to WHO grade III anaplastic and WHO grade II diffuse astrocytoma. Interestingly, there was a gradient of MCT4 expression from the leading edge to the center of the tumor. In addition, we found a similar gradient for HIF-1*α*, which is known to induce MCT4 expression under hypoxia via binding to two HRE within the *SLC16A3* promoter [15]. Of note, we identified HIF-1*α* as one of the nearest interactors of MCT4/*SLC16A3* in the STRING network analysis. The metabolic symbiosis model proposes that in an expanding heterogeneous tumor, cancer cells at the rim receive sufficient oxygen and energy supply, whereas cells in the dense tumor center are under hypoxic conditions [16]. For a long time, this has been considered a disadvantage for the tumor. However, there is mounting evidence that distinct subpopulations of cancer cells communicate with each other via the tumor microenvironment, rendering tumors even more malignant and aggressive. In this context, Sonveaux et al. suggested that hypoxic cells overexpress MCT4 to release the high amounts of lactate produced during anaerobic glycolysis into the microenvironment [16]. Lactate is preferentially taken up by oxygenated tumor cells near blood vessels via MCT1, since lactate consumption is more efficient than glycolytic enzymes involved in glucose oxidation. Lactate is converted into pyruvate in oxygenated tumor cells to fuel tricarboxylic acid cycle and oxidative phosphorylation under aerobic conditions. This mechanism enhances the glucose gradient, thereby ensuring energy supply for the hypoxic tumor regions. This metabolic circle was proposed to promote the overall growth and survival of the tumor, thereby playing a crucial role in tumor malignancy [16, 17]. In our *in silico* analysis, MCT4 was mainly upregulated in the perinecrotic and hyperplastic/microvascular proliferation zones, which represent histological malignancy criteria to distinguish WHO grade IV GBM from a WHO grade III anaplastic astrocytoma [44]. Accordingly, MCT4 overexpression was associated with shorter overall survival in GBM patients, highlighting its clinical relevance and need for further study.

**Figure 7:**
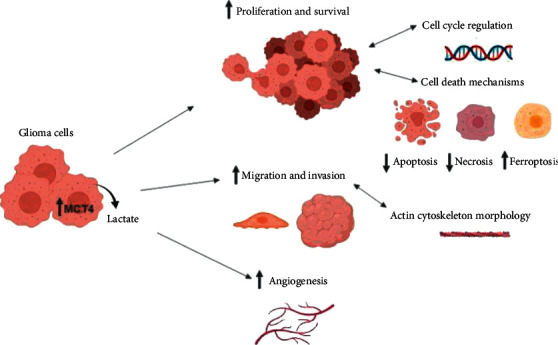
Working model of MCT4 overexpression in glioma cells. MCT4 upregulation in glioma cells increases lactate export, promoting proliferation and survival in glioma cells by modulating cell cycle regulation and cell death mechanisms. Furthermore, it leads to enhanced migration and invasiveness of glioma cells by altering actin cytoskeleton morphology. Finally, it promotes angiogenesis in surrounding blood vessels.

To investigate the functional consequences of MCT4 expression in glioma, we generated an MCT4 overexpression and knockdown/inhibition model in F98 glioma cells, which were also shown to upregulate MCT4 under hypoxia, and performed several *in vitro* cell culture assays as well as an *ex vivo* slice culture model, much closer reflecting the *in vivo* situation. The findings are summarized in a working model (Figure 7).

Consistent with its function as lactate and proton exporter, MCT4 expression correlated inversely with pH_e_ and proportionally with extracellular lactate concentration.

Importantly, MCT4 increased proliferation and survival. Cell cycle analysis revealed that MCT4 overexpressing cells were found mostly in the viable fraction, whereas MCT4 knockdown cells contained the largest proportion of apoptotic and necrotic cells. Not surprisingly, MCT4 silencing was associated with an increased apoptotic fraction in GBM neurospheres [14]. Since intracellular acidification is toxic for the cells, upregulation of MCT4 might represent a protective mechanism of cancer cells to escape cell death by exporting intracellular protons [51]. Besides apoptosis and necrosis, ferroptosis is a recently proposed iron-dependent mechanism of cell death [37]. Interestingly, our data suggest that ferroptosis plays a role in MCT4 overexpressing F98 glioma cells. We found that only these cells could be rescued by iron-chelator treatment, thereby preventing ROS production leading to ferroptosis. However, the rescue of MCT4 overexpressing cells was only partial. Other mechanisms leading to ROS production in tumor cells are likely responsible for the partial rescue of MCT4 cells. It might be important to study these mechanisms since the induction of ferroptosis might be a novel treatment option, specifically for MCT4 overexpressing tumor cells. MCT4 is widely expressed in nonneoplastic glycolytic cells such as skeletal muscle cells [8, 52] as well as in astrocytes, where astrocyte-neuron lactate transport was shown to be important for the maintenance of neuronal activity and long-term potentiation [53]. Hence, general inhibition of MCT4 would probably have disastrous consequences. Ferroptosis-based therapies might be a possibility to selectively extinguish MCT4 overexpressing cancer cells.

Besides promoting proliferation and survival by altered cell cycle and cell death mechanisms, upregulated MCT4 enhanced cell migration and invasive potential via reorganization of the actin cytoskeleton. On the one hand, enhanced intracellular pH (pH_i_) has been shown to remodel the cytoskeleton for migration and invasion [54, 55]. On the other hand, invadopodia-mediated extracellular matrix degradation as well as activation of extracellular matrix dissolving enzymes is augmented by an acidic tumor microenvironment [56–59]. Consistent with that observation, distinct subsets of matrix metalloproteinases (MMPs) were shown to accompany MCT4 overexpression in perinecrotic and hyperplastic/microvascular proliferation tumor zones. Furthermore, EMT has been proposed to play an important role during the invasion of gliomas [49, 50]. Interestingly, MCT4/*SLC16A3* was highly upregulated in GBMs with mesenchymal molecular subtype than in those with other molecular subtypes. Most EMT-associated genes were found in the hyperplastic/microvascular proliferation tumor zones. However, correlation of expression patterns was not evident in all GBM samples, and EMT-associated genes did not reveal as nearest MCT4/*SLC16A3* interactors. Therefore, MCT4 seems to play a certain role in EMT GBMs but the exact biological mechanisms behind need to be elucidated further.

Finally, lactate treatment promoted angiogenesis in HUVEC via VEGF signaling, whereas MCT4 inhibition suppressed it, indicating that MCT4 plays a crucial role in tumor-related angiogenesis. Akin to the metabolic symbiosis model, tumors are reported to interact with nearby blood vessels via lactate shuttling [60, 61]. Lactate released by glycolytic tumor cells via MCT4 was proposed to be taken up by HUVEC via MCT1, thereby supporting proangiogenic signaling via HIF-1*α* and an autocrine NF*κ*B/IL-8 pathway. Consistent with that finding, lactate has already been shown to stimulate angiogenesis through HIF-1*α*-dependent activation of VEGF/VEGFR signaling as well as through the TGF*β* pathway [29–31]. Consistently, low pH_e_ has been related to the induction of VEGF via the MAPK pathway in human glioma cells *in vitro* as well as in brain tumors *in vivo* [62, 63].

## 5. Conclusions

In the present study, we explored the functional consequences of MCT4 expression on distinct hallmarks of tumor malignancy in F98 glioma cells using *in silico* analysis, *in vitro* cell culture assays, and *ex vivo* organotypic rat brain slice culture model. MCT4 overexpression increased tumor cell proliferation and survival by altered cell cycle regulation and cell death mechanisms. Moreover, upregulated MCT4 enhanced cell migration and invasiveness of tumor cells via reorganization of the actin cytoskeleton. Finally, MCT4 inhibition mitigated the induction of angiogenesis, suggesting that MCT4 also plays a crucial role in tumor-related angiogenesis.

MCT4 is of pivotal clinical relevance in GBM, since patient survival inversely correlates with MCT4 expression levels. In a nutshell, our study provides broad functional evidence for MCT4/*SLC16A3* as a key driver for multiple hallmarks of malignancy in glioma. Moreover, this study suggests that specifically targeting MCT4 overexpressing glioma cells by the induction of ferroptosis may lead to novel approaches in GBM treatment.

## Data Availability

Data are available upon request through the authors themselves. Please contact Anna Maria Reuss, Institute of Neuropathology, University Hospital Zurich, University of Zurich, Zurich, Switzerland (annamaria.reuss@uzh.ch).

## Supplementary Materials

Supplementary Materials Figure S1: DEGs in GBM and endogenous MCT4 expression in glioma cell lines (related to Figure 1). Figure S2: MCT4 overexpression, knockdown, and inhibition in F98 glioma cells (related to Figure 2). Figure S3: invasion genes in GBM tumor compartments (related to Figure 3). Figure S4: treatment of HUVEC and native brain slices (related to Figure 4). Figure S5: gating controls for flow cytometry analyses (related to Figure 6). Figure S1: DEGs in GBM and endogenous MCT4 expression in glioma cell lines. (a) Percentage of significantly up- and downregulated genes in perinecrotic and hyperplastic/microvascular proliferation tumor zones compared to the leading edge (*n* = 135). Data were obtained from the IVY Glioblastoma Atlas Project database. (b) Relative MCT4 mRNA expression in different glioma cell lines as determined by qRT-PCR (mean ± SEM, *n* = 4). Figure S2: MCT4 overexpression, knockdown, and inhibition in F98 glioma cells. (a) MCT4 mRNA expression ratios in MCT4 F98 cells transiently knocked down with three different siRNAs and normalized to MCT4, as determined by qRT-PCR. Statistical analysis was performed by one-way ANOVA with Tukey's posttest (^*∗*^*p* < 0.05; ^*∗∗*^*p* < 0.01, mean ± SEM, *n* = 2). (b) Toxicity curve for F98 cells treated with increasing concentrations of pCMBS, as determined by MTT assay after 72 h. (c) Toxicity curve for F98 cells treated with increasing concentrations of Phl, as determined by MTT assay after 72 h. Figure S3: invasion genes in GBM tumor compartments. (a) Gene expression analysis of MCT4/*SLC16A3* and MMPs in histological GBM compartments, shown as normalized gene-level FPKM values *(n* = 278). Data were obtained from the IVY Glioblastoma Atlas Project database. (b) Gene expression analysis of MCT4/*SLC16A3* in histological GBM compartments with different molecular subtypes, shown as normalized gene-level FPKM values *(n* = 278). Data were obtained from the IVY Glioblastoma Atlas Project database. (c) Gene expression analysis of MCT4/*SLC16A3* and EMT genes in histological GBM compartments, shown as normalized gene-level FPKM values *(n* = 278). Data were obtained from the IVY Glioblastoma Atlas Project database. Figure S4: treatment of HUVEC and native brain slices. (a) Tube formation of HUVEC in 3D cell culture treated with 100 *μ*M pCMBS, 150 *μ*M Phl, or 10 mM Lac. Scale bar represents 200 *μ*m. Quantification of tube formation parameters for the assessment of angiogenesis was performed with Fiji. Data were normalized to untreated control. Statistical analysis was performed by two-way ANOVA with Bonferroni posttest (^*∗∗*^*p* < 0.01, mean ± SEM, *n* = 3). (b) Native brain slices treated with 100 *μ*M pCMBS, 150 *μ*M Phl, or 10 mM Lac and stained with PI directly after and 48 h after treatment. Quantification of cell death intensity in native brain slices was performed with Fiji. Data were normalized to untreated control (ctrl). Statistical analysis was performed by two-way ANOVA with Bonferroni posttest (^ns^*p* > 0.05; ^*∗*^*p* < 0.05, mean ± SEM, *n* = 3). (c) Blood vessel sprouting in native brain slices treated with 100 *μ*M pCMBS, 150 *μ*M Phl, or 10 mM Lac and stained with antilaminin after 5 d in culture. Quantification of blood vessel sprouting parameters for assessing angiogenesis was performed with Fiji. Data were normalized to untreated control (ctrl). Statistical analysis was performed by two-way ANOVA with Bonferroni posttest (^*∗∗*^*p* < 0.01; ^*∗∗∗*^*p* < 0.001, mean ± SEM, *n* = 3). Figure S5: gating controls for flow cytometry analyses. (a) Unstained F98 wt control for gating of GFP-negative cells. (b) Unstained F98 wt control for gating of Annexin V-Pacific Blue-negative and PI-negative cells. Table S1: siRNA sequences. Table S2: primer sequences. Table S3: antibodies and dyes.

## References

[1] Rasmussen B. K., Hansen S., Laursen R. J. (2017). Epidemiology of glioma: clinical characteristics, symptoms, and predictors of glioma patients grade I-IV in the the Danish Neuro-Oncology Registry. *Journal of Neuro-Oncology*.

[2] Davis F. G., Smith T. R., Gittleman H. R., Ostrom Q. T., Kruchko C., Barnholtz-Sloan J. S. (2019). Glioblastoma Incidence Rate Trends in Canada and the United States Compared with England, 1995–2015. *Neuro-Oncology*.

[3] Castro M. G., Cowen R., Williamson I. K. (2003). Current and future strategies for the treatment of malignant brain tumors. *Pharmacology & Therapeutics*.

[4] Cheng L., Wu Q., Guryanova O. A. (2011). Elevated invasive potential of glioblastoma stem cells. *Biochemical and Biophysical Research Communications*.

[5] Batash R., Asna N., Schaffer P., Francis N., Schaffer M. (2017). Glioblastoma multiforme, diagnosis and treatment; recent literature review. *Current Medicinal Chemistry*.

[6] Anton K., Baehring J. M., Mayer T. (2012). Glioblastoma multiforme. *Hematology/Oncology Clinics of North America*.

[7] Delgado-López P. D., Corrales-García E. M. (2016). Survival in glioblastoma: a review on the impact of treatment modalities. *Clinical and Translational Oncology*.

[8] Price N. T., Jackson V. N., Halestrap A. P. (1998). Cloning and sequencing of four new mammalian monocarboxylate transporter (MCT) homologues confirms the existence of a transporter family with an ancient past. *Biochemical Journal*.

[9] Dimmer K. S., Friedrich B., Lang F., Deitmer J. W., Broer S. (2000). The low-affinity monocarboxylate transporter MCT4 is adapted to the export of lactate in highly glycolytic cells. *Biochemical Journal*.

[10] Pinheiro C., Reis R. M., Ricardo S., Longatto-Filho A., Schmitt F., Baltazar F. (2010). Expression of monocarboxylate transporters 1, 2, and 4 in human tumours and their association with CD147 and CD44. *Journal of Biomedicine and Biotechnology*.

[11] Grillon E., Farion R., Fablet K., De Waard M., Tse C. M., Donowitz M. (2011). The spatial organization of proton and lactate transport in a rat brain tumor. *PLoS One*.

[12] Curry J. M., Tuluc M., Whitaker-Menezes D. (2013). Cancer metabolism, stemness and tumor recurrence. *Cell Cycle*.

[13] Miranda-Gonçalves V., Honavar M., Pinheiro C. (2013). Monocarboxylate transporters (MCTs) in gliomas: expression and exploitation as therapeutic targets. *Neuro-Oncology*.

[14] Lim K. S., Lim K. J., Price A. C., Orr B. A., Eberhart C. G., Bar E. E. (2014). Inhibition of monocarboxylate transporter-4 depletes stem-like glioblastoma cells and inhibits HIF transcriptional response in a lactate-independent manner. *Oncogene*.

[15] Ullah M. S., Davies A. J., Halestrap A. P. (2006). The plasma membrane lactate transporter MCT4, but not MCT1, is up-regulated by hypoxia through a HIF-1*α*-dependent mechanism^∗^. *Journal of Biological Chemistry*.

[16] Sonveaux P., Végran F., Schroeder T. (2008). Targeting lactate-fueled respiration selectively kills hypoxic tumor cells in mice. *The Journal of Clinical Investigation*.

[17] Draoui N., Feron O. (2011). Lactate shuttles at a glance: from physiological paradigms to anti-cancer treatments. *Disease Models & Mechanisms*.

[18] Kallinowski F., Vaupel P. (1988). pH distributions in spontaneous and isotransplanted rat tumours. *British Journal of Cancer*.

[19] Gerweck L. E., Seetharaman K. (1996). Cellular pH gradient in tumor versus normal tissue: potential exploitation for the treatment of cancer. *Cancer Research*.

[20] McSheehy P. M. J., Stubbs M., Griffiths J. R. (2000). Role of pH in tumor-trapping of the anticancer drug 5-fluorouracil. *Advances in Enzyme Regulation*.

[21] Gladden L. B. (2004). Lactate metabolism: a new paradigm for the third millennium. *The Journal of Physiology*.

[22] Chiche J., Brahimi-Horn M. C., Pouysségur J. (2010). Tumour hypoxia induces a metabolic shift causing acidosis: a common feature in cancer. *Journal of Cellular and Molecular Medicine*.

[23] Wike-Hooley J. L., Haveman J., Reinhold H. S. (1984). The relevance of tumour pH to the treatment of malignant disease. *Radiotherapy and Oncology*.

[24] Walenta S., Wetterling M., Lehrke M. (2000). High lactate levels predict likelihood of metastases, tumor recurrence, and restricted patient survival in human cervical cancers. *Cancer Research*.

[25] Reichert M., Steinbach J. P., Supra P., Weller M. (2002). Modulation of growth and radiochemosensitivity of human malignant glioma cells by acidosis. *Cancer*.

[26] Gatenby R. A., Gawlinski E. T., Gmitro A. F., Kaylor B., Gillies R. J. (2006). Acid-mediated tumor invasion: a multidisciplinary study. *Cancer Research*.

[27] Quennet V., Yaromina A., Zips D. (2006). Tumor lactate content predicts for response to fractionated irradiation of human squamous cell carcinomas in nude mice. *Radiotherapy and Oncology*.

[28] Mashima T., Sato S., Sugimoto Y., Tsuruo T., Seimiya H. (2009). Promotion of glioma cell survival by acyl-CoA synthetase 5 under extracellular acidosis conditions. *Oncogene*.

[29] Kumar V. B. S., Viji R. I., Kiran M. S., Sudhakaran P. R. (2007). Endothelial cell response to lactate: implication of PAR modification of VEGF. *Journal of Cellular Physiology*.

[30] Schmid S. A., Gaumann A., Wondrak M. (2007). Lactate adversely affects the in vitro formation of endothelial cell tubular structures through the action of TGF-*β*1. *Experimental Cell Research*.

[31] Xiong J., Yang Q., Li J., Zhou S. (2014). Effects of MDM2 inhibitors on vascular endothelial growth factor-mediated tumor angiogenesis in human breast cancer. *Angiogenesis*.

[32] Pinheiro C., Garcia E. A., Morais-Santos F., Moreira M. A., Almeida F. M., Jube L. F. (2015). Reprogramming energy metabolism and inducing angiogenesis: co-expression of monocarboxylate transporters with VEGF family members in cervical adenocarcinomas. *BMC Cancer*.

[33] Baek G., Tse Y. F., Hu Z. (2014). MCT4 defines a glycolytic subtype of pancreatic cancer with poor prognosis and unique metabolic dependencies. *Cell Reports*.

[34] Baenke F., Dubuis S., Brault C. (2015). Functional screening identifies MCT4 as a key regulator of breast cancer cell metabolism and survival. *The Journal of Pathology*.

[35] Ghoochani A., Yakubov E., Sehm T. (2016). A versatile ex vivo technique for assaying tumor angiogenesis and microglia in the brain. *Oncotarget*.

[36] Liu Y., Peterson D. A., Kimura H., Schubert D. (1997). Mechanism of cellular 3-(4,5-dimethylthiazol-2-yl)-2,5-diphenyltetrazolium bromide (MTT) reduction. *Journal of Neurochemistry*.

[37] Dixon S. J., Lemberg K. M., Lamprecht M. R. (2012). Ferroptosis: an iron-dependent form of nonapoptotic cell death. *Cell*.

[38] Burk R. R. (1973). A factor from a transformed cell line that affects cell migration. *Proceedings of the National Academy of Sciences*.

[39] Macpherson I., Montagnier L. (1964). Agar suspension culture for the selective assay of cells transformed by polyoma virus. *Virology*.

[40] Kubota Y., Kleinman H. K., Martin G. R., Lawley T. J. (1988). Role of laminin and basement membrane in the morphological differentiation of human endothelial cells into capillary-like structures. *Journal of Cell Biology*.

[41] Park S. J., Yoon B. H., Kim S. K., Kim S. Y. (2019). GENT2: an updated gene expression database for normal and tumor tissues. *BMC Med Genomics*.

[42] Puchalski R. B., Shah N., Miller J. (2018 05). An anatomic transcriptional atlas of human glioblastoma. *Science*.

[43] Szklarczyk D., Gable A. L., Lyon D. (2019 01). STRING v11: protein-protein association networks with increased coverage, supporting functional discovery in genome-wide experimental datasets. *Nucleic Acids Research*.

[44] Louis D. N., Perry A., Reifenberger G. (2016). The 2016 world health organization classification of tumors of the central nervous system: a summary. *Acta Neuropathologica*.

[45] Fox J. E. M., Meredith D., Halestrap A. P. (2000). Characterisation of human monocarboxylate transporter 4 substantiates its role in lactic acid efflux from skeletal muscle. *The Journal of Physiology*.

[46] Hanahan D., Weinberg R. A. (2000). The hallmarks of cancer. *Cell*.

[47] Rungger-Brandle E., Gabbiani G. (1983). The role of cytoskeletal and cytocontractile elements in pathologic processes. *The American Journal of Pathology*.

[48] Nakada M., Okada Y., Yamashita J. (2003). The role of matrix metalloproteinases in glioma invasion. *Frontiers in Bioscience*.

[49] Iser I. C., Pereira M. B., Lenz G., Wink M. R. (2017). The epithelial-to-mesenchymal transition-like process in glioblastoma: an updated systematic review and in silico investigation. *Medicinal Research Reviews*.

[50] Tao C., Huang K., Shi J., Hu Q., Li K., Zhu X. (2020). Genomics and prognosis analysis of epithelial-mesenchymal transition in glioma. *The American Journal of Pathology*.

[51] Gatenby R. A., Gillies R. J. (2004). Why do cancers have high aerobic glycolysis?. *Nature Reviews Cancer*.

[52] Halestrap A. P. (2013). The SLC16 gene family-structure, role and regulation in health and disease. *Molecular Aspects of Medicine*.

[53] Suzuki A., Stern S. A., Bozdagi O. (2011). Astrocyte-neuron lactate transport is required for long-term memory formation. *Cell*.

[54] Bernstein B. W., Painter W. B., Chen H., Minamide L. S., Abe H., Bamburg J. R. (2000). Intracellular pH modulation of ADF/cofilin proteins. *Cell Motility and the Cytoskeleton*.

[55] Magalhaes M. A. O., Larson D. R., Mader C. C. (2011). Cortactin phosphorylation regulates cell invasion through a pH-dependent pathway. *Journal of Cell Biology*.

[56] Bourguignon L. Y. W., Singleton P. A., Diedrich F., Stern R., Gilad E. (2004). CD44 interaction with Na+-H+ exchanger (NHE1) creates acidic microenvironments leading to hyaluronidase-2 and cathepsin B activation and breast tumor cell invasion. *Journal of Biological Chemistry*.

[57] Stuwe L., Muller M., Fabian A., Waning J., Mally S., Noel J. (2007). pH dependence of melanoma cell migration: protons extruded by NHE1 dominate protons of the bulk solution. *The Journal of Physiology*.

[58] Busco G., Cardone R. A., Greco M. R. (2010). NHE1 promotes invadopodial ECM proteolysis through acidification of the peri‐invadopodial space. *The FASEB Journal*.

[59] Gioia M., Fasciglione G. F., Monaco S. (2010). pH dependence of the enzymatic processing of collagen I by MMP-1 (fibroblast collagenase), MMP-2 (gelatinase A), and MMP-14 ectodomain. *JBIC Journal of Biological Inorganic Chemistry*.

[60] Végran F., Boidot R., Michiels C., Sonveaux P., Feron O. (2011). Lactate influx through the endothelial cell monocarboxylate transporter MCT1 supports an NF-*κ*B/IL-8 pathway that drives tumor angiogenesis. *Cancer Research*.

[61] Sonveaux P., Copetti T., De Saedeleer C. J., Vegran F., Verrax J., Kennedy K. M. (2012). Targeting the lactate transporter MCT1 in endothelial cells inhibits lactate-induced HIF-1 activation and tumor angiogenesis. *PLoS One*.

[62] Fukumura D., Gohongi T., Kadambi A. (2001). Predominant role of endothelial nitric oxide synthase in vascular endothelial growth factor-induced angiogenesis and vascular permeability. *Proceedings of the National Academy of Sciences*.

[63] Xu L., Fukumura D., Jain R. K. (2002). Acidic extracellular pH induces vascular endothelial growth factor (VEGF) in human glioblastoma cells via ERK1/2 MAPK signaling pathway: mechanism of low pH-induced VEGF. *Journal of Biological Chemistry*.

