# Supplementary material for: MCT4 Promotes Tumor Malignancy in F98 Glioma Cells
Source: J Oncol. 2021 Apr 14;2021:6655529. doi: 10.1155/2021/6655529 (PMC8060090; doi:10.1155/2021/6655529)
Supplement: Supplementary Materials — Figure S1: DEGs in GBM and endogenous MCT4 expression in glioma cell lines (related to Figure 1). Figure S2: MCT4 overexpression, knockdown, and inhibition in F98 glioma cells (related to Figure 2). Figure S3: invasion genes in GBM tumor compartments (related to Figure 3). Figure S4: treatment of HUVEC and native brain slices (related to Figure 4). Figure S5: gating controls for flow cytometry analyses (related to Figure 6). Figure S1: DEGs in GBM and endogenous MCT4 expression in glioma cell lines. (a) Percentage of significantly up- and downregulated genes in perinecrotic and hyperplastic/microvascular proliferation tumor zones compared to the leading edge (n = 135). Data were obtained from the IVY Glioblastoma Atlas Project database. (b) Relative MCT4 mRNA expression in different glioma cell lines as determined by qRT-PCR (mean ± SEM, n = 4). Figure S2: MCT4 overexpression, knockdown, and inhibition in F98 glioma cells. (a) MCT4 mRNA expression ratios in MCT4 F98 cells transiently knocked down with three different siRNAs and normalized to MCT4, as determined by qRT-PCR. Statistical analysis was performed by one-way ANOVA with Tukey's posttest (∗p < 0.05; ∗∗p < 0.01, mean ± SEM, n = 2). (b) Toxicity curve for F98 cells treated with increasing concentrations of pCMBS, as determined by MTT assay after 72 h. (c) Toxicity curve for F98 cells treated with increasing concentrations of Phl, as determined by MTT assay after 72 h. Figure S3: invasion genes in GBM tumor compartments. (a) Gene expression analysis of MCT4/SLC16A3 and MMPs in histological GBM compartments, shown as normalized gene-level FPKM values (n = 278). Data were obtained from the IVY Glioblastoma Atlas Project database. (b) Gene expression analysis of MCT4/SLC16A3 in histological GBM compartments with different molecular subtypes, shown as normalized gene-level FPKM values (n = 278). Data were obtained from the IVY Glioblastoma Atlas Project database. (c) Gene expression analysis of MCT4/SLC16A3 and EMT [file 6655529.f1.zip › 6655529.f1/TableS1.pdf]

siRNA	**Sequence 5’→ 3’**
Fw-siRNA#1	GATCCCCCCGTCAGTGTCTTCTTCAATTCAAGAGAT TGAAGAAGACACTGACGGTTTTTGGAAA
Fw-siRNA#2	GATCCCCCGACCCACGTCTACATGTATTCAAGAGAT ACATGTAGACGTGGGTCGTTTTTGGAAA
Fw-siRNA#3	GATCCCCCGGCAACGCTTGCTATTTATTCAAGAGAT AAATAGCAAGCGTTGCCGTTTTTGGAAA
Rv-siRNA#1	AGCTTTTCCAAAAACCGTCAGTGTCTTCTTCAATCTC TTGAATTGAAGAAGACACTGACGGGGG
Rv-siRNA#2	AGCTTTTCCAAAAACGACCCACGTCTACATGTATCTC TTGAATACATGTAGACGTGGGTCGGGG
Rv-siRNA#3	AGCTTTTCCAAAAACGGCAACGCTTGCTATTTATCTC TTGAATAAATAGCAAGCGTTGCCGGGG
